# A protocol for a network meta-analysis of interventions to treat patients with sudden sensorineural hearing loss

**DOI:** 10.1186/s13643-018-0736-3

**Published:** 2018-05-16

**Authors:** Nadera Ahmadzai, Shaun Kilty, Dianna Wolfe, Jamie Bonaparte, David Schramm, Elizabeth Fitzpatrick, Vincent Lin, Wei Cheng, Becky Skidmore, David Moher, Brian Hutton

**Affiliations:** 1 The Ottawa Hospital Research Institute, Center for Practice Changing Research, 501 Smyth Road, Box 201, Ottawa, Ontario K1H 8L6 Canada; 2 Department of ENT, The Ottawa Hospital, Ottawa, Canada; 3 The University of Ottawa School of Epidemiology, Public Health and Preventive Medicine, Ottawa, Canada; 4 Faculty of Health Sciences, University of Ottawa, Ottawa, Canada; 5 Children’s Hospital of Eastern Ontario Research Institute, Ottawa, Canada; 6 Department of Otolaryngology—Head & Neck Surgery, Sunnybrook Health Sciences Centre, Sunnybrook Research Institute, Toronto, Canada; 7 Faculty of Medicine, University of Toronto, Toronto, Canada

**Keywords:** Sensorineural hearing loss, Network meta-analysis, Systematic review

## Abstract

**Background:**

Hearing loss is one of the leading causes of disability worldwide, with greater than 20% of Canadian adults having measurable hearing loss in at least one ear. Patients with hearing loss experience impaired quality of life, and emotional and financial consequences that affect themselves and their families. Sudden sensorineural hearing loss (SSNHL) is a common but difficult to treat form of hearing loss that has a sudden onset of ≤ 72 h associated with various etiologies, with the majority of cases being idiopathic. Some patients may partially or completely recover hearing ability, but for 32 to 65% of patients whose hearing does not recover, feelings of social isolation elevate the risk of anxiety and depression. Hearing loss is also associated with poorer functional status, including difficulty with sound localization and hearing in noise. There exists a wide range of therapeutic options; however, treatment of idiopathic SSNHL is controversial because some patients recover spontaneously. The planned systematic review and network meta-analysis (NMA) will assess the relative effects of competing treatments for management of idiopathic SSNHL in adults.

**Methods:**

Electronic search strategies were developed by an experienced medical information specialist in consultation with the review team. We will search MEDLINE, Embase, and the Cochrane Library with no date or language restrictions. Key clinical trial registries will also be searched for in-progress and completed trials. Two reviewers will independently screen the literature using pre-specified eligibility criteria, and assess the quality of included studies using the Cochrane Risk of Bias tool. Disagreements will be resolved through consensus or third party adjudication. Bayesian NMAs will be pursued to compare interventions in terms of their effects on hearing (including audiometric thresholds and speech recognition scores), extent of hearing recovery, quality of life, and incidence of harms (including vestibular dysfunction, incidence of infections, and withdrawals due to adverse events).

**Discussion:**

This systematic review and NMA will offer new and informative evaluations of current therapies for SSNHL. The results will inform clinicians as to the relative benefits of the currently available interventions for managing this difficult condition, provide optimal clinical treatment strategies, establish evidence gaps, and identify promising treatments for evaluation in future trials.

**Systematic review registration:**

PROSPERO registration number: CRD 42017073756.

## Background

Hearing loss is one of the leading causes of disability worldwide [1], with > 20% of adult Canadians having measurable hearing loss in at least one ear [2]. While many interventions are available, Canadians with hearing loss endure a severely impaired quality of life [3]. Sudden sensorineural hearing loss (SSNHL) can be particularly disruptive, often occurring in the prime of mid-life. The emotional and financial toll of SSNHL on patients, their families, and society is large and often underestimated. Early detection and rapid intervention are advised [4]. However, selection of appropriate treatments among the therapies available can be difficult because of the variety of etiologies of hearing loss and the uncertainty surrounding the efficacy of various interventions.

SSNHL is a common but difficult to treat type of hearing loss. It is a debilitating condition with major impacts on mental health and quality of life. Approximately 27 new cases of SSNHL develop per 100,000 persons annually in the USA [5]. SSNHL occurs rapidly over 72 h or less (≤ 3 days) [6, 7], often with other incapacitating symptoms, including tinnitus, intractable vertigo, and hyperacusis, resulting in extreme patient anxiety [8, 9]. While some individuals may recover some or all hearing, for the 32 to 65% of patients who do not [10], the social isolation associated with the inability to understand speech, the inability to localize sound, and pervasive tinnitus lead to increased risk of anxiety disorder and depression [11–13]. Etiologies are numerous; however, the majority of cases of SSNHL are idiopathic [8, 14]. Often when patients present in a primary care setting, SSNHL is not immediately investigated due to the prioritization of other potential causes of hearing loss ahead of SSNHL, such as hearing loss secondary to an upper respiratory tract infection [15]. Suggested therapeutic options for SSNHL are diverse, and include anti-inflammatories, antiviral agents, diuretics, vasodilators, rheologic agents, tri-iodobenzoic acid derivatives, and surgical interventions. However, the ideal treatment of idiopathic SSNHL remains controversial due to the potential for spontaneous recovery in many patients [10]. For patients who receive no or inappropriate treatment and who do not spontaneously recover, the lifelong social isolation and work-related difficulties associated with chronic single-sided deafness and tinnitus remain [13]. These difficulties are exponential for individuals with pre-existing hearing loss in their other ear. The detrimental effects of SSNHL on patients’ quality of life are severe. There is a need to establish with greater certainty the benefits of previously studied interventions, as well as to prioritize the available treatment interventions and to identify considerations for future research.

While traditional pairwise meta-analyses of direct evidence are of great value and familiarity to researchers, physicians, and decision makers, they cannot address comparisons of multiple interventions in a cohesive analysis. Network meta-analysis is a vital methodology available to address situations where multiple comparators of relevance exist. This protocol describes methodology for a systematic review and network meta-analysis that will assess the relative effects of competing treatments for management of idiopathic SSNHL in terms of hearing recovery, pure tone audiometry, speech recognition scores, quality of life, reduction of tinnitus, vestibular endpoints, and harms.

## Methods

This protocol was developed in consultation with the Preferred Reporting Items for Systematic Review Protocols (PRISMA-P) Statement [16], and is registered with the International Prospective Register of Systematic Reviews (PROSPERO) database (CRD#42017073756). Any protocol modifications made during the conduct of the review will be described in the publication of the final report. The PRISMA Extension Statement for NMA will be followed to guide preparation of the final report to ensure all aspects of methods and findings are reported [17].

### Data sources and search for studies

An experienced information specialist developed a preliminary search strategy in consultation with the review team. The literature search will be conducted in MEDLINE, Embase, and the Cochrane Library, with no date or language restrictions. Searches will utilize a combination of controlled vocabulary (e.g., “Hearing Loss, Sensorineural,” “Hearing Loss, Sudden”) and keywords (e.g., SSNHL, sudden deafness, sudden sensorineural hearing loss) and a randomized controlled trial (RCT) filter that will be applied in MEDLINE and Embase. Syntax will be adjusted according to the needs of each database. The search will be peer reviewed prior to execution by a second information specialist using the PRESS Criteria [18] and appropriate recommendations incorporated. We will perform a separate search for systematic reviews to compare the list of included studies from existing reviews against those retrieved from the core RCT searches. A targeted gray literature search of ClinicalTrials.gov and the International Clinical Trials Registry Platform search portal will also be undertaken to identify in-progress and completed trials.

The MEDLINE search strategy is presented in Appendix.

### Study eligibility criteria

Eligibility criteria for the review have been designed according to the PICOS (Population-Intervention-Comparators-Outcomes-Study design) framework. We will include studies that meet the following criteria:

#### Population

Adult patients with idiopathic single-sided SSNHL, defined as a 30 dB hearing loss in three consecutive frequencies in one ear whose onset occurs in ≤ 3 days, with cause being disruption of the cochlea of the inner ear, the vestibular nerve or higher regions of auditory processing [5]. Cases of binaural and non-idiopathic single-sided SSNHL will not be included; such cases are usually associated with underlying conditions and should be managed in accordance [6].

#### Interventions/comparators

To maintain homogeneity across studies, the review will focus on first line therapy, excluding salvage therapy. The following interventions will be of interest: *systemic steroids* (dexamethasone, hydrocortisone, betamethasone, prednisone, cortisone, prednisolone, methylprednisolone); *antivirals* (valacyclovir, acyclovir); *systemic volume expansion* (hydroxyethyl starch); *anti*-*thrombotics* (pentoxifylline, batroxobin, recombinant tissue plasminogen activator); *vasodilators* (prostaglandin E 1, naftidrofuryl); *increased tissue oxygenation therapies* (carbogen); *hyperbaric oxygen therapy*; *intratympanic steroid*-*combination therapies* (intratympanic methylprednisolone, intratympanic dexamethasone); *anti*-*inflammatory therapies* (Dextran 40, Dextran 40 + procaine hydrochloride); *anti*-*platelet vasodilatation* (prostacyclin, pentoxifylline); *other therapies* [magnesium aspartate, magnesium sulphate, fibrinogen/LDL apheresis, mannitol, nifedipine, fludiazepam, diazepam, hyperbaric oxygen, vitamin A, vitamin E, zinc, Chinese herbal medicine, *Ginkgo biloba* extract, AM-111 (a c-Jun N-terminal Kinase (JNK) ligand), Ozone therapy (auto-haemotherapy)]. The listed interventions have been structured into broad categories; primary analyses will consider interventions at the group level, with the exception of those listed in the “Other therapies” category, where additional treatment nodes may be used. We will also explore the feasibility of analyses at a more granular level to avoid the assumption of class effects. Following data collection, our clinical experts will be consulted to establish whether additional treatment groupings are needed to maximize the representativeness of unique interventions (such as considering variable doses, durations, or strengths of steroid therapy). If combination therapies are encountered, they will be included as additional treatment nodes. We will clearly identify any post-hoc, alternative network geometries formed using this approach in the final review.

#### Outcomes

Endpoints of interest will include hearing measures assessed via audiometric tests [i.e., pure tone audiometry, speech recognition scores (including word recognition and speech discrimination scores, and speech reception threshold (SRT)], and via clinical tests (i.e., tuning fork by Rinner test, and Weber test) where available; extent of recovery (e.g., Sieigel’s Criteria, or other such endpoint measures that categorize patients’ recovery as complete, marked, slight, or none based on decibels of improved hearing); quality of life (generic and disease-specific measures); reduction of tinnitus [could be measured via different techniques such as psychoacoustic tests of tinnitus (e.g., pitch match, loudness match, maskability, residual inhibition), rating scales (e.g., verbal rating scale, numerical rating scale, visual analog scale, poster style, mechanical device, etc.), questionnaires describing functional effects (e.g., tinnitus questionnaire, tinnitus handicap questionnaire, tinnitus severity scale, subjective tinnitus severity scale/tinnitus reaction questionnaire, tinnitus severity grading, tinnitus severity index, tinnitus handicap inventory, intake interview for tinnitus retraining therapy), and patients’ global perception of treatment-related changes]; incidence of vestibular endpoints (e.g., vertigo); and harms (e.g., withdrawals due to adverse effects, otitis media, residual tympanic membrane perforation).

#### Study design

Randomized controlled trials of any duration will be included.

### Screening and data extraction

We will perform screening in two stages via four reviewers working in pairs independently and in duplicate against a priori eligibility criteria using an online systematic review software program (Distiller Systematic Review (DSR) Software; Evidence Partners Inc., Ottawa, Canada). Screening at stage 1 will encompass review of titles and abstracts identified from the electronic search, while stage 2 will be based upon review of full text articles of those deemed potentially relevant during stage 1. We will start screening at both stages with a calibration exercise to ensure consistent application of eligibility criteria. Disagreements among reviewers will be resolved through consensus or third party adjudication. A PRISMA flow diagram [19] will be prepared to document the study selection process in the final publication. The list of included studies of the existing reviews will be inspected to confirm no relevant studies are missed.

A standardized form implemented in Microsoft Excel (Microsoft Corporation, Seattle, Washington, USA) will be used for data extraction, recording key items. Data extraction will be performed by one reviewer and verification will be carried out by a second reviewer. We will extract the following information from each study: publication characteristics, including authors, publication year, and journal; study design details (e.g., cited trial design, clinical setting, duration of follow up, number of patients randomized and number analyzed for each outcome, occurrence of dropouts, funding source, and authors’ conflict of interest); study population characteristics (e.g., patient eligibility criteria, age, sex, BMI, race, comorbidities, and other relevant baseline data, such as PTA, prior otologic surgery); intervention and comparator characteristics, including type (e.g., systemic steroids, antivirals, anti-thrombotic, etc.), dose, unit, duration, frequency, route of administration, and co-intervention; and outcome data, including reported outcome definitions and summary data related to treatment effects (e.g., mean or mean difference and SD for continuous outcomes, and numbers of events and number of total patients for dichotomous outcomes), and reported tools/scales used to evaluate outcomes.

### Risk of bias assessment

The Cochrane Risk of Bias Tool for RCTs [20] will be used to evaluate the risk of bias of each included RCT. Assessments will be carried out by two reviewers independently, and disagreement will be resolved via consensus or third party adjudication. The domains of the Cochrane risk of bias tool for RCTs that will be assessed include selection bias (sequence generation, and allocation sequence concealment), performance bias (blinding of participants and personnel), detection bias (blinding of outcome assessment), attrition bias (incomplete outcome data), reporting bias (selective reporting), and other biases (other source of bias). To assess baseline imbalances between groups, we will consider comorbidities that may predispose to hearing loss, including the following: history of upper respiratory tract infection, benign paroxysmal positional vertigo, vertebrobasilar insufficiency, posterior inferior cerebellar artery syndrome, basilar migraine, cerebellar disease, multiple sclerosis, tumors of brainstem and fourth ventricle, epilepsy, cervical vertigo, and Meniere’s disease.

### Approach to evidence synthesis

**Fig. 1:**
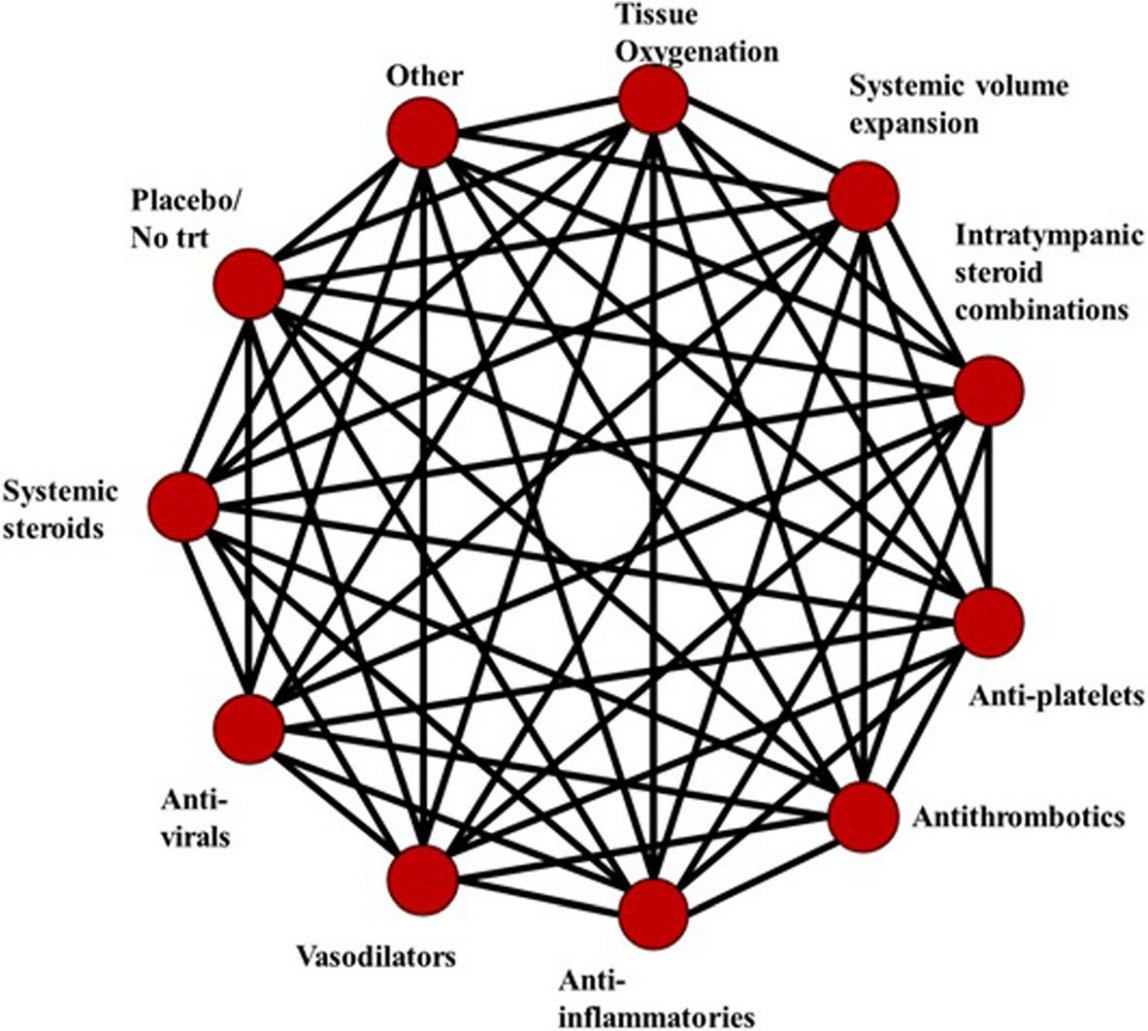
Primary network for SSNHL (group level). A preliminary depiction of the group-level network structure is shown. The extent to which the network’s treatments will be connected will be dependent upon the body of literature identified in the systematic review

Characteristics of included studies, including patients’ clinical characteristics (e.g., age, sex, clinical history of key factors including duration of impairment, baseline severity, etc.) and methodologic homogeneity (e.g., risk of bias), will be inspected and summarized. Meta-analyses will be performed if studies are judged to be sufficiently homogeneous by members of the research team, and the transitivity assumption found to be appropriate. This decision will be informed by careful consideration of the collected patient characteristics (e.g., eligibility criteria and demographics such as baseline PTA and history of prior otologic surgery, etc.) and study methods (e.g., setting, follow-up, etc.) by the research team. We will also perform pairwise meta-analyses for each comparison of interventions (at the group level) using the available studies to quantify statistical heterogeneity using the I^2^ measure, which will inform further explorations of heterogeneity between studies; if heterogeneity is judged to be excessive, a narrative summary with supporting tables and figures to present findings will be employed. If the homogeneity of studies is sufficient, we will perform fixed and random effects Bayesian NMAs to compare interventions contained within the included studies. Bayesian NMAs will use a common heterogeneity parameter as per established methods [21–23]. We will assess model fit by comparing residual deviance with the number of unconstrained data points [24]; model fit will be considered adequate if these quantities are approximately equal. The selection between models will be based on deviance information criteria (DIC), with a difference of five points suggesting an important difference [24]. The use of specific NMA models will be determined by the type of endpoint under analysis (e.g., continuous or binary). Generally, mean differences (MD) are used to compare continuous endpoints measured in the same units. If we come across continuous endpoints that are measured using different scales across studies (e.g., a visual analog scale from 0 to 100 versus an itemized, composite score scale to assess severity of vertigo attacks), we will consider a model for standardized mean differences (SMD) to explore benefits across related scales and maximize available data. If we apply SMD for analyses of an outcome, we will explore the use of established methods to present results in minimal important difference (MID) units such that findings are more interpretable [25, 26]. Summary estimates for binary endpoints will be expressed as odds ratios. All pairwise comparisons between interventions will be expressed with 95% credible intervals. The consistency of direct and indirect evidence will be assessed by fitting unrelated means models and comparing their DIC with that from the corresponding consistency model (with differences of 5 points or more indicating an important difference in fit), and scatterplots of deviance residuals derived from both models will also be assessed. If potential indications of inconsistency are encountered, we will explore study characteristics which may explain their appearance, and explore sensitivity analyses (e.g., adjustments or exclusions) to address the issue. All NMAs will be performed using OpenBUGS version 3.2.3 (http://openbugs.net). Model convergence will be assessed using Gelman Rubin diagnostics and inspection of Monte Carlo errors. To assess the impact of covariates on our findings, we will explore subgroup analyses and/or meta-regression adjustments [22, 27] chosen in collaboration with our clinical experts; these will include (but not be limited to) gender distribution (e.g., % males), age, prevalence of dizziness, patients’ prior history of steroid use, number of days since onset of hearing loss (or time to treatment), and severity of initial hearing loss; any characteristics added after protocol identification will be identified as post-hoc analyses in the final review. Description of the structure of the treatment network at the group level for the review is presented in Fig. 1 (though not all pairwise comparisons may be informed by trial data as shown, dependent upon the comparisons present in the final set of included studies). We will estimate key secondary measures of effect, namely SUCRA and average treatment rankings [28], to explore potential orderings of treatment hierarchy. Comparison-adjusted funnel plots [29] will be used to explore for the presence of publication bias.

## Discussion

A variety of pharmaceuticals have been used in treating SSNHL including systematic steroids, antivirals, systemic volume expansion, anti-thrombotics, vasodilators, increased tissue oxygenation therapies, intratympanic steroid-combination therapies, anti-inflammatory therapies, anti-platelet vasodilatation, and other therapies. Traditional systematic reviews exist that have compared effectiveness of certain pairs of interventions in patients with SSNHL [10, 14, 30]; however, no NMAs enabling comparison of the multiplicity of interventions in a unified synthesis and making use of direct and indirect evidence have been performed [31–34]. NMA enables researchers to address more clinically relevant questions by considering all clinically relevant comparators and incorporating all available direct and indirect evidence [31, 32]. This planned review incorporating NMA will offer new and informative evaluations of current therapies for SSNHL and enhance insights into the relative benefits of the available interventions for managing this difficult condition.

We will publish the results of this review in a clinical specialty journal with the intent of maximizing outreach to physicians pursuing prospective research for this condition. This effort will extend to emergency and primary care physicians, who in some cases may be the first to encounter this patient group. The report will present evidence networks summarizing past studies as well as ongoing studies (identified from www.clinicaltrials.gov and other registries) to establish the current state of the evidence base and assess its ongoing evolution. Consideration of network geometry in light of the findings from NMAs can be of value to researchers and funders intent on avoiding waste of research funds on trials evaluating treatment comparisons of limited value [35]. In addition to a peer-reviewed publication, we will also draft lay summaries to post online and for distribution to key societies and patient groups.

## Appendix

### Search strategy

#### RCTs

Database: Ovid MEDLINE(R) Epub Ahead of Print, In-Process & Other Non-Indexed Citations, Ovid MEDLINE(R) Daily and Ovid MEDLINE(R) <1946 to Present> Search Strategy:

1. exp. hearing loss, sensorineural/
2. Hearing Loss, Sudden/
3. sudden*.tw,kw.
4. 1 and (2 or 3)
5. Hearing Loss, Sudden/
6. (sudden* adj3 ((loss* or lose or losing) adj2 hear*)).tw,kw.
7. (sudden* adj3 ((degrad* or deteriorat*) adj2 hear*)).tw,kw.
8. (sudden* adj3 (impair* adj2 hear*)).tw,kw.
9. (sudden* adj3 deaf*).tw,kw.
10. (sudden* adj3 (hypacusia* or hypacus?s or hypoacousia or hypoacus?s)).tw,kw.
11. (SSHL or SSNHL or ISSHL or ISSNHL or SISHL or SISNHL).tw,kw.
12. (SSHLs or SSNHLs or ISSHLs or ISSNHLs or SISHLs or SISNHLs).tw,kw.
13. Hearing Loss/
14. sudden*.tw,kw.
15. 13 and 14
16. or/4-12,15
17. exp. Animals/ not (exp Animals/ and Humans/)
18. 16 not 17
19. (comment or editorial or interview or news).pt.
20. (letter not (letter and randomized controlled trial)).pt.
21. 18 not (19 or 20)
22. (controlled clinical trial or randomized controlled trial or pragmatic clinical trial).pt.
23. clinical trials as topic.sh.
24. Randomized Controlled Trials as Topic/
25. (randomi#ed. or randomi#ation* or randomly or RCT$1 or placebo*).tw,kw.
26. ((singl* or doubl* or trebl* or tripl*) adj (mask* or blind* or dumm*)).tw,kw.
27. trial.ti.
28. or/22-27
29. 21 and 28 [RCTs]

#### Reviews

Database: Ovid MEDLINE(R) Epub Ahead of Print, In-Process & Other Non-Indexed Citations, Ovid MEDLINE(R) Daily and Ovid MEDLINE(R) <1946 to Present> Search Strategy:

1. exp. hearing loss, sensorineural/
2. Hearing Loss, Sudden/
3. sudden*.tw,kw.
4. 1 and (2 or 3)
5. Hearing Loss, Sudden/
6. (sudden* adj3 ((loss* or lose or losing) adj2 hear*)).tw,kw.
7. (sudden* adj3 ((degrad* or deteriorat*) adj2 hear*)).tw,kw.
8. (sudden* adj3 (impair* adj2 hear*)).tw,kw.
9. (sudden* adj3 deaf*).tw,kw.
10. (sudden* adj3 (hypacusia* or hypacus?s or hypoacousia or hypoacus?s)).tw,kw.
11. (SSHL or SSNHL or ISSHL or ISSNHL or SISHL or SISNHL).tw,kw.
12. (SSHLs or SSNHLs or ISSHLs or ISSNHLs or SISHLs or SISNHLs).tw,kw.
13. Hearing Loss/
14. sudden*.tw,kw.
15. 13 and 14
16. or/4-12,15
17. exp. Animals/not (exp Animals/and Humans/)
18. 16 not 17
19. (comment or editorial or interview or news).pt.
20. (letter not (letter and randomized controlled trial)).pt.
21. 18 not (19 or 20)
22. limit 21 to systematic reviews
23. meta analysis.pt.
24. exp. meta-analysis as topic/
25. (meta-analy* or metanaly* or metaanaly* or met analy* or integrative research or integrative review* or integrative overview* or research integration or research overview* or collaborative review*).tw,kw.
26. (systematic review* or systematic overview* or evidence-based review* or evidence-based overview* or (evidence adj3 (review* or overview*)) or meta-review* or meta-overview* or meta-synthes* or “review of reviews” or technology assessment* or HTA or HTAs).tw,kw.
27. exp. Technology assessment, biomedical/
28. (cochrane or health technology assessment or evidence report).jw.
29. (network adj (MA or MAs)).tw,kw.
30. (NMA or NMAs).tw,kw.
31. indirect comparison?.tw,kw.
32. (indirect treatment* adj1 comparison?).tw,kw.
33. (mixed treatment* adj1 comparison?).tw,kw.
34. (multiple treatment* adj1 comparison?).tw,kw.
35. (multi-treatment* adj1 comparison?).tw,kw.
36. simultaneous comparison?.tw,kw.
37. mixed comparison?.tw,kw.
38. or/23-37
39. 21 and 38
40. 22 or 39 [SYSTEMATIC REVIEWS]

## Abbreviations

MD: Mean difference
MID: Minimal important difference
NMA: Network meta-analysis
PICO: Population, intervention, comparator, outcome
PRESS: Peer review of electronic search strategies
PTA: Pure tone audiometry
SMD: Standardized mean difference
SR: Systematic review
SRT: Speech reception threshold
SSNHL: Sudden sensorineural hearing loss
